# Supplementary material for: Electronic and Molecular Adsorption Properties of Pt-Doped BC6N: An Ab-Initio Investigation
Source: Nanomaterials (Basel). 2024 Apr 26;14(9):762. doi: 10.3390/nano14090762 (PMC11085478; doi:10.3390/nano14090762)
Supplement: Supplementary file 1 [file nanomaterials-14-00762-s001.zip › nanomaterials-2933379-supplementary.pdf]

Supplementary Information (SI)

Electronic and Molecular Adsorption Properties of Pt-doped BC_6N: An Ab-initio Investigation

Nada M. Alghamdi[1], Mohamed M. Fadlallah[2],*

Hind M. Al-qahtani[3], and Ahmed A. Maarouf[4†]

[1] *Department of Physics, College of Science,*

Imam Abdulrahman Bin Faisal University, Dammam 31441, Saudi Arabia

[2] *Physics Department, Faculty of Science,*

Benha University, Benha 13518, Egypt

[3] *Department of Physics, College of Science and Humanities,*

Imam Abdulrahman Bin Faisal University, Jubail 3196, Saudi Arabia and

[4] *Department of Physics, Faculty of Basic Sciences,*

German University in Cairo, New Cairo City 11835, Egypt

* mohamed.fadlallah@fsc.bu.edu.eg

† ahmed.maarouf@guc.edu.eg

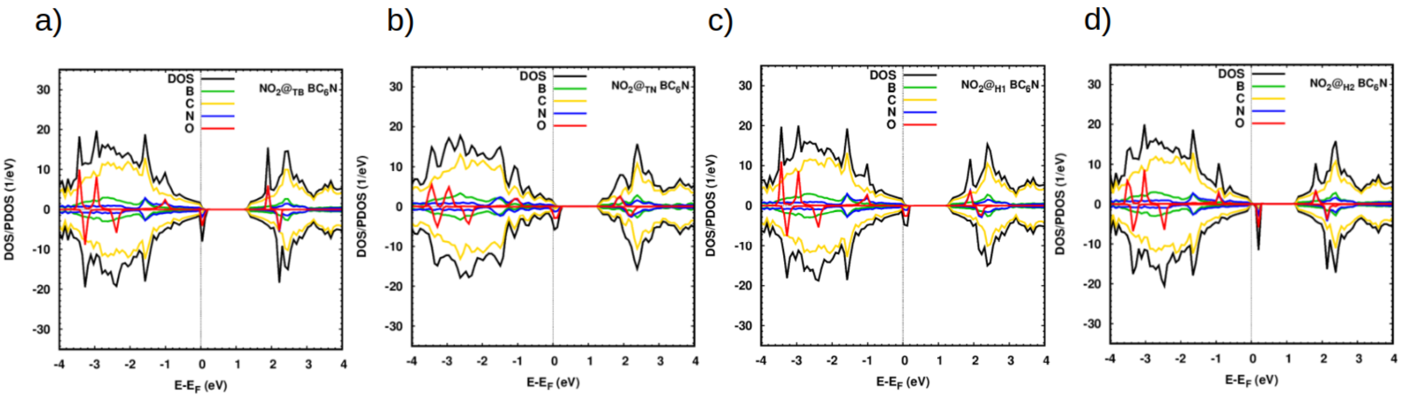

FIG. S 1. DOS/projected DOS (PDOS) of: (a) $NO_2@_{TB}BC_6N$, (b) $NO_2@_{TN}BC_6N$, (c) $NO_2@_{H1}BC_6N$ and (d) $NO_2@_{H2}BC_6N$.

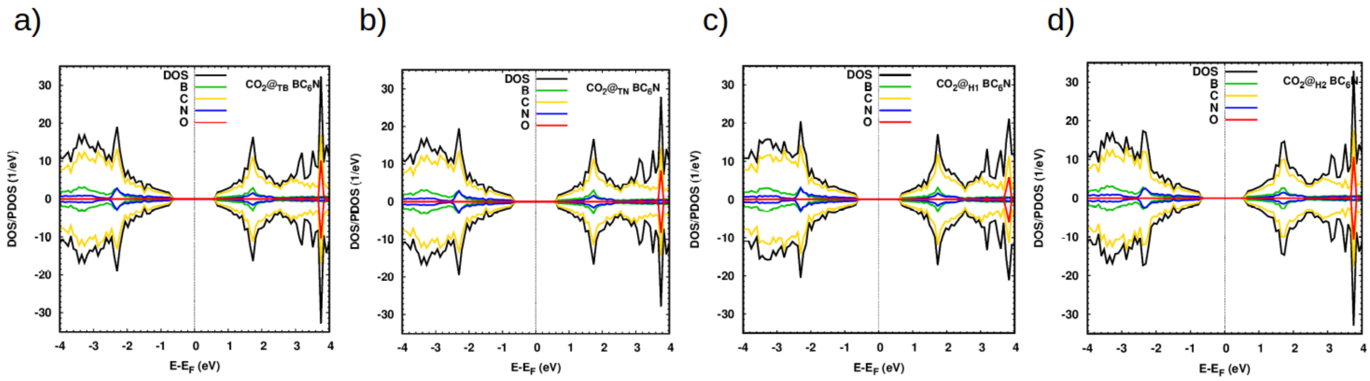

FIG. S 2. DOS/projected DOS (PDOS) of: (a) $CO_2@_{TB}BC_6N$, (b) $CO_2@_{TN}BC_6N$, (c) $CO_2@_{H1}BC_6N$ and (d) $CO_2@_{H2}BC_6N$.

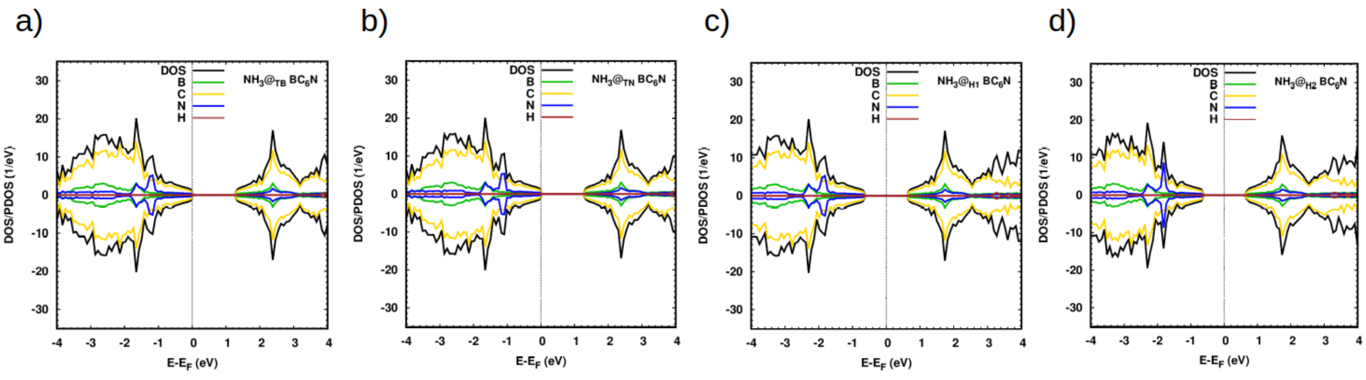

FIG. S 3. DOS/projected DOS (PDOS) of: (a) $NH_3@_{TB}BC_6N$, (b) $NH_3@_{TN}BC_6N$, (c) $NH_3@_{H1}BC_6N$ and (d) $NH_3@_{H2}BC_6N$.
